# Antiproliferative, Cytotoxic, and Apoptotic Activity of Steroidal Oximes in Cervicouterine Cell Lines

**DOI:** 10.3390/molecules21111533

**Published:** 2016-11-14

**Authors:** Luis Sánchez-Sánchez, María Guadalupe Hernández-Linares, María L. Escobar, Hugo López-Muñoz, Edgar Zenteno, María A. Fernández-Herrera, Gabriel Guerrero-Luna, Alan Carrasco-Carballo, Jesús Sandoval-Ramírez

**Affiliations:** 1 Facultad de Estudios Superiores Zaragoza, Universidad Nacional Autónoma de México, 09230 Ciudad de México, Mexico; luisss@unam.mx (L.S.-S.); hugogris@hotmail.com (H.L.-M.); 2 Laboratorio de Investigación, Jardín Botánico Universitario, Benemérita Universidad Autónoma de Puebla, 72570 Puebla, Pue., Mexico; gabriel.guerrero@alumno.buap.mx (G.G.-L.); alan.carballo@alumno.buap.mx (A.C.-C.); 3 Departamento de Biología Celular, Facultad de Ciencias, Universidad Nacional Autónoma de México, 04510 Ciudad de México, Mexico; escobarluisa@ciencias.unam.mx; 4 Departamento de Bioquímica, Facultad de Medicina, Universidad Nacional Autónoma de México, 04510 Ciudad de México, Mexico; ezenteno@unam.mx; 5 Centro de Investigación UNAM-UABJO, 68120 Oaxaca, Oax., Mexico; 6 Departamento de Física Aplicada, Centro de Investigación y de Estudios Avanzados—Unidad Mérida, km 6 Antigua Carretera a Progreso, Cordemex, 97310 Mérida, Yuc., Mexico; mfernandez@cinvestav.mx; 7 Facultad de Ciencias Químicas, Benemérita Universidad Autónoma de Puebla, 72570 Puebla, Pue., Mexico; jesus.sandoval@correo.buap.mx

**Keywords:** steroidal oximes, 23-acetyldiosgenin, (25*R*)-spirost-4-en-3,6-dione, apoptosis, antiproliferative activity

## Abstract

Steroidal sapogenins have shown antiproliferative effects against several tumor cell lines; and their effects on human cancer cells are currently under study. Changes in the functionality on the steroidal structure make it possible to modify the biological activity of compounds. Herein, we report the synthesis and in vitro antitumor activity of two steroidal oxime compounds on cervical cancer cells. These derivatives were synthesized from the steroidal sapogenin diosgenin in good yields. The in vitro assays show that the steroidal oximes show significant antiproliferative activity compared to the one observed for diosgenin. Cell proliferation, cell death, and the cytotoxic effects were determined in both cervical cancer cells and human lymphocytes. The cancer cells showed apoptotic morphology and an increased presence of active caspase-3, providing the notion of a death pathway in the cell. Significantly, the steroidal oximes did not exert a cytotoxic effect on lymphocytes.

## 1. Introduction

**Figure 1:**
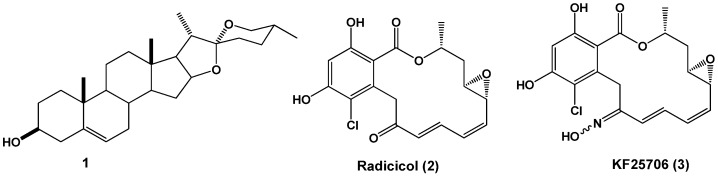
Diosgenin (**1**); radicicol (**2**); and radicicol oxime derivative (**3**).

Cancers figure among the leading causes of morbidity and mortality worldwide, with therapies based primarily on surgery, radiation therapy, and chemotherapy which, to date, are not successful interventions, and the complete removal of the cancer without damage to other tissues is the ideal goal of treatment. Sometimes this can be accomplished by surgery, but the propensity of cancers to invade adjacent tissues or to spread to distant sites by microscopic metastasis often limits its effectiveness. On the other hand, chemotherapy and radiotherapy may have a negative effect on normal cells [1]. Antitumor research is a very active field, and a large amount of information dealing with clinical aspects of cancer chemotherapy is generated; there is, however, a continuing need for new treatments inspired by medicinal chemistry and drug design. In this field, synthetic chemistry has been broadly employed to modify drug targets, especially those of natural origin, in order to find a solution to the problem of the limited supply of natural products by developing synthetic strategies. Sapogenins constitute a class of natural products that occur widely in their glycoside form and promote diverse health properties; among them the steroidal sapogenins (spirostans) are the most important bioactive compounds from several plants. Many biological functions of steroidal sapogenins have been reported, e.g., anticarcinogenic, antithrombotic, antiviral, hypocholesterolemic, and hypoglycemic [2,3,4,5,6,7,8,9,10,11,12,13,14,15,16,17,18,19,20,21]. Diosgenin (**1**, Figure 1) is one of the most available steroidal sapogenins; it was first isolated by Takeo Tsukamoto in 1936 from *Dioscorea tokoro* [22]. Spirostan **1** is the major constituent in fenugreek seeds (*Trigonella foenum-graecum* L.) and in wild yams (*Dioscorea villosa* L.) which are consumed as food ingredients or condiments by some populations in Latin America, Eastern Europe, and Asia [23,24].

Diosgenin (**1**) is used in the pharmaceutical industry as the main precursor in the synthesis of steroids [25]. It has the ability to penetrate cell membranes and bind to specific receptors [26]. However, mammals are unable to convert **1** into important steroidal metabolites, such as cholesterol, pregnanes, androstanes, etc., due to the lack of the appropriate enzymes involved in steroid hormone biosynthesis. In that sense, there is no evidence to validate the claim of an estrogenic potency of diosgenin in humans [24,25,27]. The trustworthiness of its consumption by humans has been established [28]. The beneficial effect of diosgenin (**1**) on human health has so far been limited to its efficacy in preventing metabolic diseases like hypercholesterolemia [29]. However, it was recently found that 1 exerts antiproliferative activity against cell lines; as HeLa (cervical cancer) [30], HEL and K562 (erythroleukemia) [31], osteosarcoma 1547 [32], HepG2, C3A, and HUH-7 (hepatocellular carcinoma) [33], and MCF-7 (breast cancer) [34] among others. Some studies have determined that diosgenin is also an apoptosis inducer and may act as a chemopreventive agent [11]. In addition, many naturally occurring and synthetic derivatives with an oxime moiety became important because of their broad biological activity profile: anti-inflammatory, antifungal, antibacterial, anticancer, or antiviral [35,36]. For example, radicicol (**2**) shows antitumor activity in in vivo assays, but radicicol oxime derivatives (**3**) exhibit a higher antitumor activity in both in vivo and in vitro assays [37,38] (Figure 1).

This fact has stimulated the search for new routes to synthesize steroidal derivatives with optimal biological activity from this pharmacophore on a large scale. In 2009, Cui et al. reported the synthesis and cytotoxic activity of a series of oximes derived from a cholestane, campestane, and stigmastane skeletons [39,40]. They suggested that the presence of the oxime group on ring B, a hydroxyl group on ring A or B, and a cholesterol-type side chain, resulted in high cytotoxicity when evaluated against a diversity of cancer cells such as Sk-Hep-1, H-292, PC-3, and Hey-1B [41]. In this work, we modified the spirostane side chain inserting the hydroxyimino group in the A/B rings (C-3 and C-6) and in the side chain (at C-23). The ketones, as precursors of α, β-unsaturated oximes, as well as the oxime esters, showed important cytotoxic activity in various cancer cell lines [42,43]. Herein, we report the synthesis of the spirostan oximes **4** and **5** and the evaluation of their antiproliferative, apoptotic, and necrotic activity in cervical cancer cells and human peripheral blood lymphocytes.

## 2. Results and Discussion

### 2.1. Synthesis

**Scheme 1:**
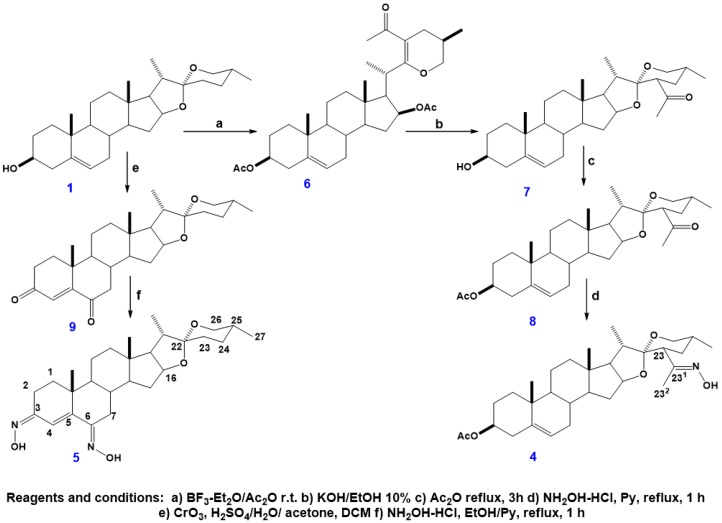
Synthesis of oxime of (23*R*)-acetyldiosgenin acetate (**4**) and dioxime of (25*R*)-spirost-4-en-3,6-dione (**5**).

The strategy to synthesize the 23-hydroxyimino derivative **4** was based on the introduction of an acetyl group at C-23 of sapogenin **1** and a further oximation of the (23*R*)-23-acetyldiosgenin acetate by treatment with NH_2_OH·HCl. The preparation of oximes at A/B rings was accomplished in two steps; the strategy was to obtain the diketone **9** by oxidation of 1 with Jones Reagent, and to generate the dioxime **5** derivative by treatment with hydroxylamine hydrochloride, as described in Scheme 1.

### 2.2. NMR Analysis

**Figure 2:**
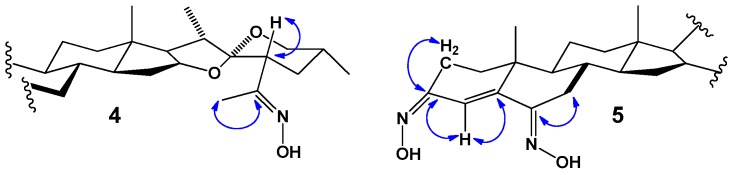
HMBC correlations for the oxime (**4**) and dioxime (**5**).

The structure of products was confirmed by 2D NMR and HRMS analyses (see Supplementary Data). Analysis of the ^1^H-NMR spectrum of **4** showed the axial proton H-23 at 2.62 ppm as a doublet of doublets presenting coupling constants with protons H-24 (*J_23a,24a_* = 12.4 Hz, *J_23a,24e_* = 5.8 Hz). The axial proton H-23 of **8** is located at 2.51 ppm, a similar position for the same proton in compound **7**. The ^13^C spectrum of **4** showed the C-23^1^ at 159.6 ppm is different from the C-23^1^ in **8**, which is located at 209.1 ppm, and that these two effects result from the formation of the hydroxylamine moiety; the data were confirmed with the HMBC experiment (Figure 2).

The NMR spectra show a single set of signals for oxime **4**. The stereochemistry of the oxime **4** was corroborated by a NOESY experiment, which shows that **4** has the hydroxyl group in an *anti* position with regard to the alkyl residue. Furthermore, the C-23^2^ is in the same plane as the H in the C-23 position.

Analysis of the ^1^H-NMR spectrum of **5** showed a single signal at 6.58 ppm for H-4, which was shifted downfield compared to the H-4 of **9**, observed at 6.18 ppm. In the ^13^C-NMR of **5** two signals around 156 ppm corresponded to C-3 and C-6, the carbon atoms of the oximes. These signals are up-field compared with those of carbonyls at the derivative **9** (201.8 ppm and 199.4 ppm). The HMBC experiment showed that H-4 correlated with the carbons 3 and 6. Other correlations in the HMBC experiment between C-3 with and H-2, and C-6 with H-7 are found. Furthermore, correlations among H-17, H-20, H-21, and H-23 with C-22 are clearly exhibited (Figure 2).

For the synthesis of **4**, several synthetic routes are available for the large-scale preparation of 23-acetylsapogenins, i.e., in a previous work, we reported the preparation of **7** through the hydrolysis of the 22,26-epoxycholestane **6** (Scheme 1), which is obtained from **1** [44] by the treatment with Ac_2_O and Et_2_O·BF_3_, at r.t., followed by quenching with water. Subsequently, the (25*R*,23*R*)-acetyldiosgenin acetate (**8**) was obtained in excellent yield through a standard acetylation. Next, the oximation of **8** using NH_2_OH·HCl afforded the expected 23-hydroxyimino derivative **4**. This structural addition of nitrogen at the spirostane side chain, promoted important changes for the higher bioactivity in comparison to other structural motifs as observed in the literature. The oxime function in the steroidal framework has been studied by several authors and first isolated from marine sponges [41,45]. In different reports, the (6*E*)-hydroxyimino derivatives exhibited a selective cytotoxic activity against several types of cancer cells [46,47,48]. Furthermore, this group of steroids was also reported to show a high affinity for human placental aromatase and to function as a competitive inhibitor of this enzyme [48]. The structural features, which appear to be important for the higher bioactivity in comparison to other structural motifs, is the existence of a ketone functionality at C3, and an elevated degree of oxidation in ring A [49]. The configuration of the oxime does not represent an influence on cytotoxic activity since it has shown similar activity patterns [50]. Thus, we produced the dioxime derivative **5** through the oximation of the 3,6-diketone derivative **9**. The synthesis of oximes **4** and **5** was conceived to evaluate them in cervical cancer cell lines and analyze their antiproliferative activity and cytotoxicity in both cellular types: cancer cells and normal cells (lymphocytes).

### 2.3. Biological Activity

#### 2.3.1. Antiproliferative Activity on HeLa and CaSki Cervical Cancer Cell Lines

**Figure 3:**
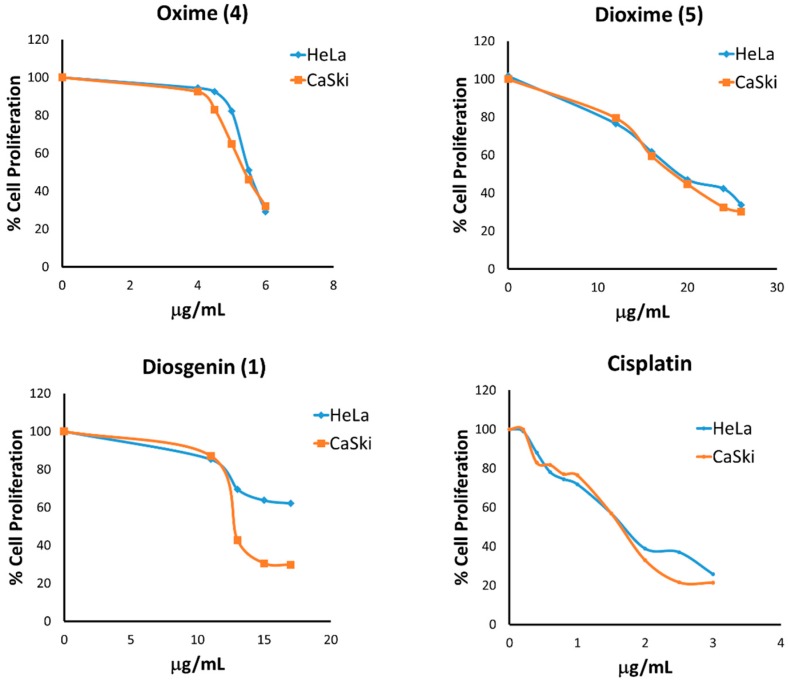
Dose-response curves of the antiproliferative effect of compounds **1**, **4**, and **5** on cervicouterine cancer cell lines HeLa and CaSki. Cisplatin was used as positive control.

In order to determine the antiproliferative activity of steroidal compounds, diosgenin (**1**) was dissolved in EtOH:DMSO 3:1 and compounds **4** and **5** were dissolved in DMSO, and screened at a range of concentrations against cervicouterine cancer cells HeLa and CaSki. Their antiproliferative activity as measured by a decrease in cell population (IC50) was determined after 24 h by crystal violet staining and the dose-response curves are shown in Figure 3 (Table 1).

The inhibitory effect of steroidal oximes **4** and **5** on the proliferation of HeLa and CaSki cells occurred in a dose-dependent manner. Diosgenin (**1**) presented IC_50_ values between 15 and 13 μg/mL, while those for the oximes were between 5.3 and 5.6 μg/mL for compound **4**; and between 19 and 22 μg/mL for compound **5** (Table 1). These results suggest that the antiproliferative activity of **1** increased 2.3–2.8 times due to the oxime group present in the spiroketalic side chain in **4**; while the oxime groups present on the steroidal A/B rings decreased antiproliferative activity 1.2–1.7 times. Interestingly, in the literature we notice that the double bond between positions C4 and C5 on the steroidal ring A, confers a negative cytotoxic effect on the cancer cells for 3-hydroxyimino-substituted compounds, in contrast with double bond in C5–C6 [35]. These data suggest that the results in antiproliferative activity may be mediated at least to some extent, by a different behavior between steroidal oximes **4** and **5**, related to the conformational difference on the rings A/B (a double bond shift).

#### 2.3.2. Determination of Cytotoxic Activity on Cervicouterine Cancer Cells

**Figure 4:**
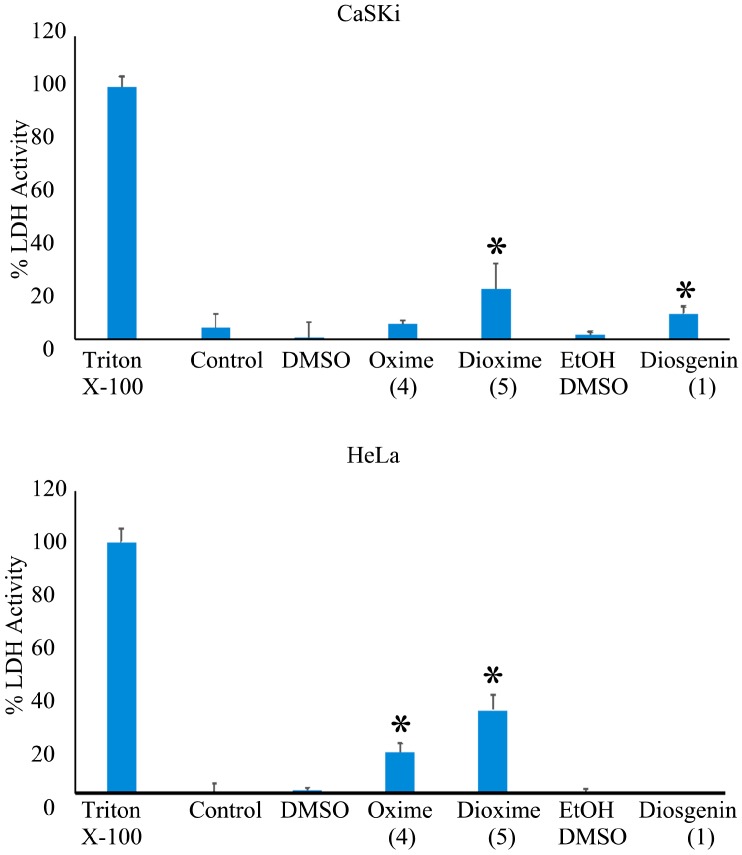
Evaluation of cytotoxicity for compounds **1**, **4**, and **5** on cervicouterine cancer cell lines. Cells were seeded in 96-well tissue culture plates at 7500 cells/well. After 24 h the medium was removed and cells were exposed to compounds **1**, **4**, and **5** at the respective IC_50_ concentrations or with EtOH/DMSO 3:1 or DMSO (10 μL/mL) and evaluated after 24 h by the amount of LDH released in the culture supernatant. Experimental data are described as mean ± S.D. of three independent experiments with three repetitions * *p* < 0.05 versus ethanol or DMSO (Student’s *t*-test).

In order to determine whether compounds **1**, **4**, and **5** induce necrosis, their cytotoxic activity was evaluated (Figure 4). Considering that the loss of the integrity of the cytoplasmic membrane is a hallmark of necrotic cell death [51], both HeLa and CaSki cell cultures were stimulated with the previously determined IC_50_ values. The amount of lactate dehydrogenase (LDH) released into the culture supernatant was used as a measure of the loss of plasma-membrane integrity. The positive control consisted of treating the cells with Triton X-100 to induce cell membrane disruption.

The two tumor cell lines HeLa and CaSki were induced with Triton X-100 in independent experiments, and the LDH released was adjusted to 100% as a control. The cytotoxicity values for compounds **1** and **4** were less than, or equal to, 15%, while for compound **5** they were higher at 20%–30%. Taken together, these findings indicate that the cytotoxic activity of compound **1** increased with the incorporation of the oxime group present in compound **5**. In addition, these results suggest that the decrease observed in the cell populations of the cultures treated with the different compounds—diosgenin **1**, monooxime **4**, or dioxime **5**—is affected through a cell death pathway distinct from necrosis, and another event different from necrosis decreases the cell number observed in cultures.

#### 2.3.3. Apoptotic Effect on Tumor Cells

Under normal conditions, cells are controlled by a balanced cell cycle such that they proliferate, lapse after a certain time, and then die, in order to maintain histological homeostasis. Apoptosis is an important, well-controlled form of cell death that is observed under a variety of physiological and pathological conditions. Unlike necrotic cell death, apoptosis does not induce an inflammatory response, thus allowing cells to be removed without producing side effects. As mentioned above, inducing apoptosis in tumor cells has become an important therapeutic target [52,53]. It is well known, for example, that inducing cells to cell-cycle arrest constitutes one of the most prevalent strategies used to stop or limit cancer spreading [54,55,56,57,58,59,60,61,62]. The decrease in the evaluated cultures suggests that compounds **4**–**5** presumably affect the cell-cycle in one of the phases (checkpoints), causing cell death. This is a matter worthy of in depth study in future work.

#### 2.3.4. Apoptotic Bodies-DAPI Staining

**Figure 5:**
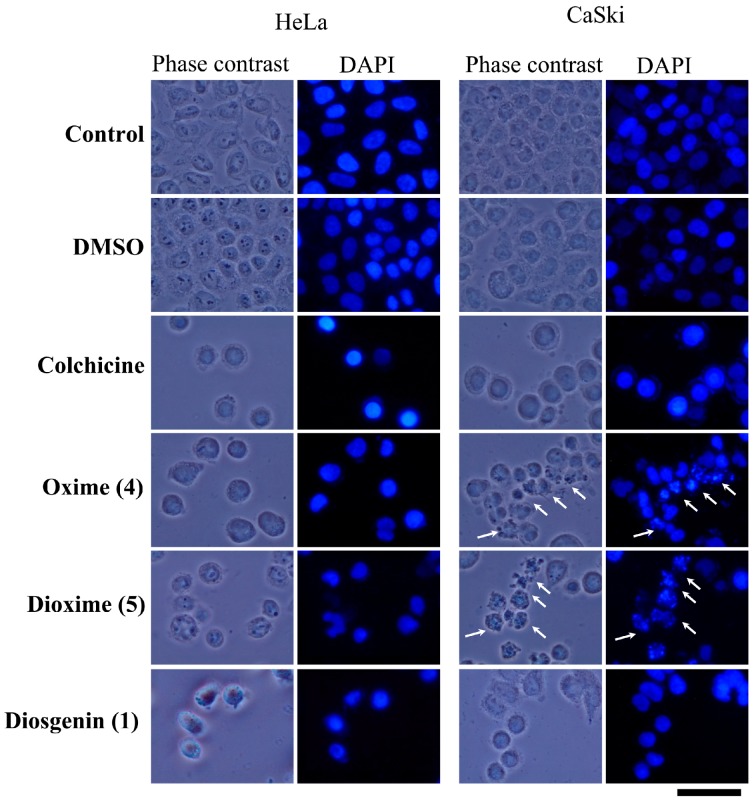
Apoptotic morphology induced by **1**, **4**, and **5** compounds on cervical cancer HeLa and CaSki cell lines. Phase contrast allows identifying the normal polyhedral morphology in control and vehicle treated cells; the normal chromatin is evident with the DAPI staining. Colchicine treated cells (positive control to apoptosis) have a spherical shape, with cytoplasmic and nuclear compaction. Compounds **1**, **4**, and **5** cause cell morphological changes corresponding to the apoptosis morphology. Apoptotic bodies are more evident in **4** and **5** than in **1** (arrows). Scale bar 50 microns.

Apoptosis is characterized by morphological changes that include cytoplasmic and chromatin condensation that produces smaller, more compact nuclei than those present in non-apoptotic cells. Highly-compacted chromatin is a key feature of apoptotic bodies and a hallmark of the apoptotic process. To evaluate the capacity of compounds **1**, **4**, and **5** to induce apoptotic cell death, HeLa and CaSki cell cultures were stimulated with them. Morphological changes and chromatin condensation, including the formation of apoptotic bodies, were identified by staining with fluorochrome 4,6-diamidino-2-phenylindole (DAPI) [63], and recognized under epifluorescence microscopy (Figure 5). Results showed that the HeLa and CaSki cells treated with compound **1** changed their morphology, showing decreased size and a spherical shape. Additionally, the nuclei had condensed chromatin, but the presence of clearly-identifiable apoptotic bodies was not confirmed. Similarly, compounds **4** and **5** caused cell contraction, but also induced DNA fragmentation and generated clear apoptotic bodies.

#### 2.3.5. Detection of Active Caspase-3

**Figure 6:**
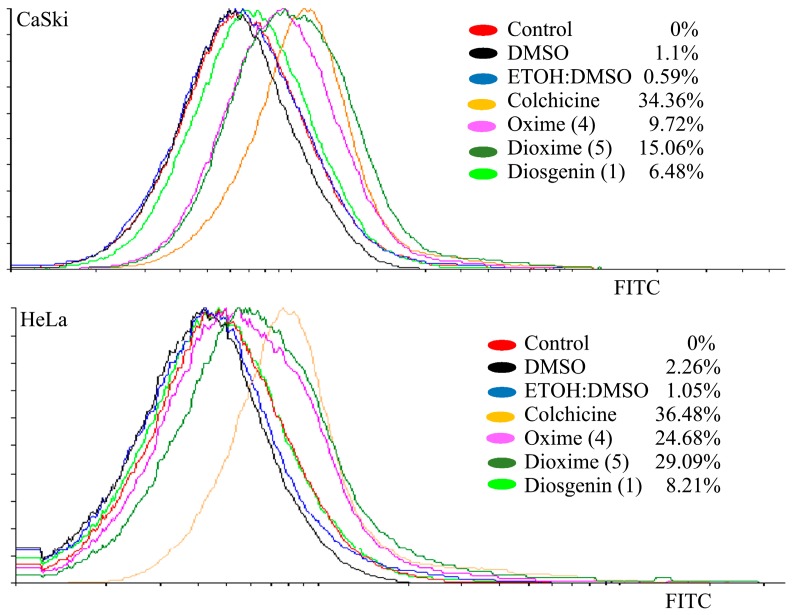
Induction of active caspase-3 by compounds **1**, **4**, and **5** on HeLa and CaSki cell cultures.

Apoptotic cell death is a programmed process that is dependent on proteases called caspases. These are a family of cysteine proteases that are crucial mediators in the process of apoptosis. Caspase-3 is perhaps the best understood of the mammalian caspases in terms of its specificity and role in apoptosis. It is also required for some typical hallmarks of apoptosis; for example, it is indispensable for apoptotic chromatin condensation and DNA fragmentation, and plays an essential role in certain processes associated with the formation of apoptotic bodies [64]. While this morphological evidence of the apoptotic process is well-documented, it is still necessary to detect the activation of effector caspases, such as active caspase-3, to establish the apoptotic process. In this study, we detected active caspase-3 by flow cytometry in order to obtain quantitative results. Our findings revealed the presence of active caspase-3 in the HeLa and CaSki cell cultures treated with all compounds (Figure 6), indicating that they induce the activation of this caspase and, therefore, that apoptosis could be triggered through a caspase-dependent process. In addition, these results, together with the effect on cell morphology, where only compound **1** induced apoptotic morphology without fragmented nuclei (i.e., fragmented chromatin), suggest that compounds **4** and **5** may have the capacity to increase the apoptotic effect of **1**. In the present work, we evaluated the antiproliferative activity of steroidal oxime derivatives; however, it is not the aim of this study to describe aspects of the molecular mechanisms that regulate the observed antitumor effects. We only addressed the potential molecular basis of these interactions, recent literature provides plausible pathways for the antitumor action of various steroidal oxime derivatives [65,66,67,68,69]. Likewise, previous reports have proposed diverse molecular mechanisms of diosgenin (**1**) to induce the cell death that include caspase-3 activation, PARP cleavage, DNA fragmentation, and cell shrinking [70]. Our results show that compounds **4** and **5** induce morphological changes such as cell shrinking and DNA compaction. Considering that active caspase-3 is present after the treatment with compounds **4** and **5**, we can hypothesize that these compounds trigger a molecular mechanism to induce apoptosis that is mediated by the activation of caspase-3 and this, in turn, activates cytoplasmic DNAase, which is responsible for DNA fragmentation.

#### 2.3.6. Evaluation of Antiproliferative Activity on Non-Tumoral Cells

**Figure 7:**
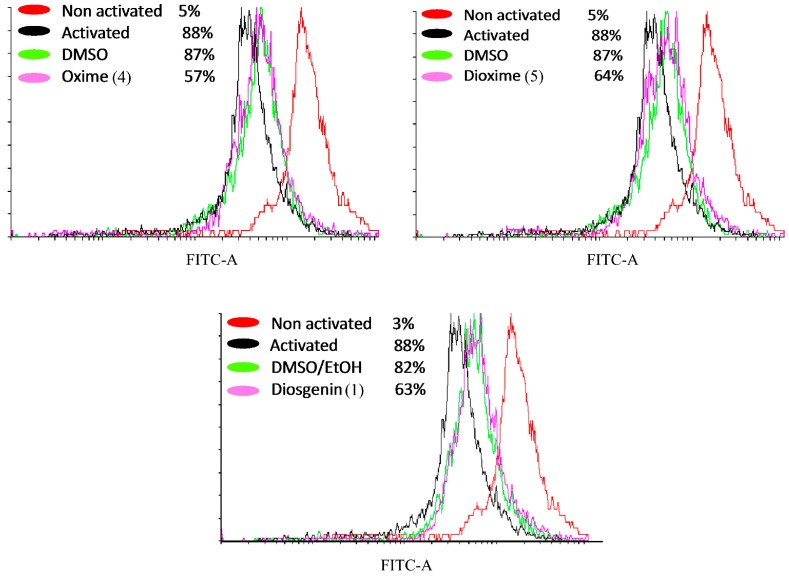
Antiproliferative activity of **1**, **4**, and **5** compounds on human lymphocyte cell cultures at the respective concentrations (**1** (15 g/mL), **4** (5.6 g/mL), and **5** (22 g/mL)).

Several compounds used currently in chemotherapy present problems for selective activity towards malignant cells and produce undesirable secondary effects. It is crucial to determine the selectivity of tested compounds using the antiproliferative and cytotoxic assays in order to derive any conclusions on the potential for anti-cancer treatment. For this reason, the effect of **1**, **4**, and **5** on the proliferation of peripheral blood lymphocytes was assessed. Lymphocytes were exposed to compounds **1** (15 g/mL), **4** (5.6 g/mL), and **5** (22 g/mL). It is well known that during chemotherapy the immune system is usually affected; thus, the proliferation of an enriched lymphocyte population (ELP) was evaluated with compounds **1**, **4**, and **5**. ELPs from a normal blood donors were labeled with 5(6)-carboxyfluorescein diacetate *N*-succinimidyl ester (CFSE), stimulated with phytohaemagglutinin (PHA), or treated with compounds **1**, **4**, or **5** and cultured for 72 h. After treatment, the cells were harvested, analyzed by flow cytometry (FACS ARIA-II, BD, San Jose, CA, USA), and the data were processed by FACSDiva 4.0 software (BD, Piscataway, NJ, USA). Considering that lymphocytes were treated longer with compounds for longer (72 h), the effect of the compounds on the proliferative potential of ELPs (Figure 7) indicates that they all slightly affected the proliferative potential of lymphocytes. Compound **1** decreased the proliferation of lymphocytic cells by 23%, while the decreases for compounds **4** and **5** were 35% and 27%, respectively. These results suggest a greater degree of antiproliferative selectivity towards malignant cell lines than lymphocytes.

#### 2.3.7. Determination of Cytotoxic Activity on Lymphocyte Cells

**Figure 8:**
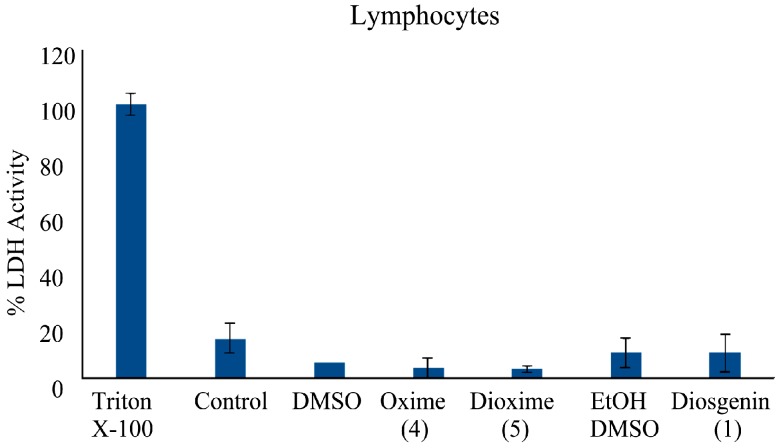
Evaluation of cytotoxicity for compounds **1**, **4,** and **5** on human lymphocyte cell cultures.

In order to determine whether necrosis is induced by these sapogenins in non-tumor cells, their cytotoxicity on human lymphocytes was evaluated (Figure 8). The application of this model is very important since the results obtained allowed us to determine lower toxicity and, hence, properly demonstrate the antiproliferative activity with minor secondary effects. The lymphocytes were activated with phytohaemagglutinin (PHA) and stimulated at the level of the IC_50_ values determined for compounds **1**, **4**, and **5**. Results indicated that these concentrations were not cytotoxic for human lymphocytes, which suggests that these steroidal oximes were not cytotoxic in non-tumor cells, so the decrease in cell populations was caused by a mechanism distinct from necrosis.

Surprisingly, lower cytotoxicity values were found in all cases, even with regard to the cytotoxicity on non-tumoral cells (human lymphocytes). These results suggest that the observed cell decrease in treated cultures is not a necrotic process.

## 3. Materials and Methods

### 3.1. General Procedures and Materials

Melting points were determined on a melt-temp apparatus at 24 °C by the open capillary method and are uncorrected. Optical rotations were measured at 24 °C in a Perkin-Elmer 241 polarimeter (Perkin Elmer Instruments LLC., Beaconsfield, UK) in chloroform solutions. ^1^H- and ^13^C-NMR spectra were recorded at 400 and 100 MHz in CDCl_3_, respectively, on a Varian MERCURY NMR instrument (Palo Alto, CA, USA). The ^1^H-NMR spectra were referenced to residual protonated solvent and for ^13^C-NMR the central signal of the triplet of CDCl_3_. Chemical shifts are given in ppm (δ-scale) and coupling constants are expressed in Hertz (Hz). All assignments were confirmed with the aid of DEPT, COSY, HSQC, HMBC and NOESY experiments. Spectra processing was performed using MestReNova software (10.0, Santiago de Compostela, Spain). High resolution mass spectra were obtained by the ESI technique using a JEOL JMS AX-505 HA mass spectrometer. IR spectra were recorded on an Agilent Cary 630 FT-IR spectrophotometer (range: 4000–600 cm^−1^). Column chromatography was performed using Merck silica gel 60 μm (230–400 mesh) by flash chromatography and analytical TLC was performed on aluminum plates pre-coated with silica gel 60F-254. The preparation of compounds **6** and **7** (Scheme 1) was performed according to the literature [44].

### 3.2. Chemical Synthesis and Characterization

#### 3.2.1. 23-Acetyldiosgenin Acetate **8**

A solution of *23-acetyldiosgenin* **7** (1.0 g, 2.19 mmol) in acetic anhydride (10 mL) was stirred at reflux for a period of 3 h. After that time, the crude product was poured into cold water and the precipitate filtered. The crude product was purified by column chromatography using a mixture of hexane/EtOAc (8:2) as eluent, to afford compound **8** as a white solid (1.04 g, 95%). m.p. 171–172 °C. [α]_D_ = −135° (*c* = 0.2). υ_max_ 2920, 1732, 1678, 1636, 1033. ^1^H-NMR (CDCl_3_) δ: 5.23 (1H, d, *J* = 5 Hz, H-6), 4.46 (1H, m, H-3), 4.28 (1H, m, H-16), 3.36 (1H, m, H-26a); 3.28 (1H, dd, *J*_26e,26a_, *J*_26e,25a_ = 11 Hz, H-26e), 2.51 (1H, dd, *J* = 12.00 Hz, H-23a), 2.28 (1H, ddd, *J*_24a,24e_ = 13 Hz, *J*_24a,23a_ = 13 Hz, *J*_24a,25e_ = 5 Hz, H-24a), 2.06 (3H, s, 23^2^), 1.89 (3H, s, CH_3_COO-3), 0.89 (3H, s, CH_3_-19), 0.85 (3H, d, *J*_21,20_ = 6.0 Hz, CH_3_-21), 0.73 (3H, d, *J*_27,25_ = 6.0 Hz, CH_3_-27), 0.54 (3H, s, CH_3_-18). ^13^C-NMR (CDCl_3_) δ: 14.1 (C-21), 16.0 (C-18), 16.9 (C-27), 19.2 (C-19), 20.6 (C-11), 21.3 (CH_3_COO-3), 27.6 (C-23^2^), 28.4 (C-24), 29.6 (C-6), 31.0 (C-7), 31.4 (C-2 and C-15), 31.9 (C-1 and C-12), 36.5 (C-8), 36.8 (C-5), 37.9 (C-9), 38.2 (C-13), 39.5 (C-20), 40.3 (C-24), 49.7 (C-25), 54.7 (C-23), 56.1 (C-14), 61.0 (C-17), 65.8 (C-26), 73.5 (C-3), 80.8 (C-16), 107.6 (C-22), 121.8 (C-6), 139.3 (C-5), 169.9 (CH_3_COO-3), 209.4 (C-23^1^).

#### 3.2.2. Oxime of 23-Acetyldiosgenin Acetate **4**

Hydroxylamine chlorhydrate (0.108 g, 1.53 mmol) was added to a solution of **8** (1.0 g, 1.92 mmol) in pyridine (30 mL) and allowed to stir at reflux. After a period of 1 h organic extraction was performed (CH_2_Cl_2_, 2 × 10 mL), the organic phase was washed with distilled water (2 × 15 mL), dried with Na_2_SO_4_ and concentrated under reduced pressure. Compound **4** was obtained (1.01 g, 93%) as a white solid, recrystallized from CH_2_Cl_2_. m.p. 180–181 °C (CH_2_Cl_2_). [α]_D_ = −24° (*c* = 0.3). υ_max_ 3309, 1732, 1666, 1636, 949. ^1^H-NMR (CDCl_3_) δ: 5.34 (1H, d, *J* = 5.6 Hz, H-6), 4.58 (1H, m, H-3) 4.38 (1H, m, H-16), 3.48 (1H, dd, *J*_26a,26e_ = 14 Hz, *J*_26a,25_ = 4 Hz, H-26a); 3.38 (1H, dd, *J*_26e,26a_ = 14 Hz, H-26e), 2.62 (1H, dd, *J*_23a,24a_ = 12.4 Hz, *J*_23a,24e_ = 5.8 Hz, H-23a), 2.31(2H, m, H-4), 2.17 (1H, dd, *J* = 12.4 Hz, H-24a), 2.02 (3H, s, CH_3_-23^2^), 1.90 (3H, s, CH_3_COO-3), 1.60 (1H, dd, *J* = 5.8 Hz H-24e), 1.00 (3H, s, CH_3_-19), 0.94 (3H, d, *J* = 6.0 Hz, CH_3_-21), 0.82 (3H, d, *J* = 6.0 Hz, CH_3_-27), 0.67 (3H, s, CH_3_-18). ^13^C-NMR (CDCl_3_) −δ: 11.9 (CH_3_-COO-3), 14.1 (C-21), 16.1 (C-18), 17.0 (C-27), 19.3 (C-19), 20.8 (C-11), 21. 5 (C-23^2^), 27.7 (C-24), 30.4 (C-8), 31.2 (C-9), 31.7 (C-1), 32.1 (C-2), 32.4 (C-10), 36.7 (C-7), 36.9 (C-15), 37.7 (C-4), 38.1 (C-13), 39.7 (C-20), 40.4 (C-24), 45.6 (C-23), 49.8 (C-25), 56.3 (C-14), 61.1 (C-17), 66.1 (C-26), 73.8 (C-3), 81.0 (C-16), 109.7 (C-22), 122.1 (C-6), 139.4 (C-5), 159.3 (C-23^1^), 170.3 (CH_3_COO-3). HRMS-EI: [M + H]^+^ calcd. for C_31_H_47_O_5_N, 513.3454; found: 513.3445.

#### 3.2.3. (25*R*)-Spirost-4-en-3,6-dione **9**

Jones Reagent (10 mL) was added dropwise at −10 °C to a solution of **1** (1.0 g, 2.41 mmol) in CH_2_Cl_2_ (40 mL) and acetone (132 mL). The temperature of the reaction mixture should not exceed 10 °C. At the end of the reaction some drops of isopropanol were added and the stirring continued, at room temperature, for 3 h, then filtered through florisil. The crude product was neutralized with saturated aqueous solution of NaHCO_3_, and the organic phase was washed with brine (2 × 50 mL) and distilled water (2 × 50 mL). The organic phase was dried with Na_2_SO_4_, concentrated under reduced pressure, and finally purified on a silica gel column, with hexane/EtOAc (8:2) as eluent, to afford **9** (0.87 g, 85%) as a white solid. m.p. 193–194 °C. [α]_D_ = −106.2° (*c* = 0.0014). υ_max_ 2949, 1685. ^1^H-NMR (CDCl_3_) δ: 6.18 (1H, s, H-4), 4.43 (1H, m, H-16), 3.48 (1H, m, H-26e), 3.36 (1H, dd, *J*_26a,26e_ = *J*_26a,25a_ = 10.4 Hz, H-26a), 2.71 (2H, d, *J*_7,8_ = 12.0 Hz, H-7), 1.19 (3H, s, CH_3_-19), 0.99 (3H, d, *J*_21,20_ = 6.8 Hz, CH_3_-21), 0.84 (3H, s, CH_3_-18), 0.80 (3H, d, *J*_27,25_ = 6.0 Hz, CH_3_-27). ^13^C-NMR (CDCl_3_) δ: 36.6 (C-1), 33.9 (C-2), 199.4 (C-3), 125.5 (C-4), 160.6 (C-5), 201.8 (C-6), 32.1 (C-7), 35.1 (C-8), 53.7 (C-9), 38.6 (C-10), 20.8 (C-11), 39.6 (C-12), 40.3 (C-13), 55.6 (C-14), 31.6 (C-15), 80.6 (C-16), 61.9 (C-17), 17.3 (C-18), 16.3 (C-19), 41.6 (C-20), 14.5 (C-21), 109.2 (C-22), 31.3 (C-23), 28.7 (C-24), 30.2 (C-25), 66.8 (C-26), 17.1 (C-27).

#### 3.2.4. Dioxime of (25*R*)-Spirost-4-en-3,6-dione **5**

To a solution of **9** (1.70 g, 4.00 mmol) in ethanol (30 mL) and pyridine (1 mL), NH_2_OH·HCl (0.560 g, 8.00 mmol) was added. The reaction mixture was stirred at reflux for 1 h. The organic phase was separated, washed with distilled water (2 × 15 mL), dried with Na_2_SO_4_ and concentrated under reduced pressure. The reaction crude was purified on silica gel column, with hexane/EtOAc (6:4) as eluent, to afford **5** (1.41 g, 77%) as white crystals, Mp 150–152 °C, [α]_D_ = +37.8° (*c* = 0.0067), υ_max_ 3313, 1681. ^1^H-NMR (CDCl_3_) δ: 6.58 (1H, s, H-4), 4.43 (1H, m, H-16), 3.48 (1H, dd, *J* = 9.2, *J* = 2.8 Hz, H-26e), 3.37 (1H, dd, *J* = 11.2, *J* = 11.2 Hz, H-26a), 3.07 (2H, dd, *J*_2,2_ = 18, *J*_2,1_ = 3.6 Hz H-2), 3.44 (2H, m, H-7), 1.06 (3H, s, CH_3_-19), 0.98 (3H, d, *J*_21,20_ = 6.8 Hz, CH_3_-21), 0.82 (3H, s, CH_3_-18), 0.79 (3H, d, *J*_27,25_ = 6 Hz, CH_3_-27). ^13^C-NMR (CDCl_3_) δ: 39.3 (C-1), 18.7 (C-2), 156.4 (C-3), 119.3 (C-4), 147.3 (C-5), 156.6 (C-6), 46.0 (C-7), 32.7 (C-8), 49.8 (C-9), 38.8 (C-10), 21.1 (C-11), 33.4 (C-12), 40.3 (C-13), 61.8 (C-14), 31.4 (C-15), 80.6 (C-16), 56.3 (C-17), 16.2 (C-18), 19.0 (C-19), 41.6 (C-20), 14.4 (C-21),109.3 (C-22), 31.3 (C-23), 28.7 (C-24), 30.2 (C-25), 66.9 (C-26), 17.1 (C-27). HRMS-EI: [M + H]^+^ calcd. for C_27_H_40_O_4_N_2_, 456.2988; found: 456.2982.

### 3.3. Biological Evaluation

#### 3.3.1. Cell Cultures

HeLa and CaSki cell lines were purchased from the American Type Culture Collection (ATCC, Rockville, MD, USA) and cultured in RPMI-1640 medium (GIBCO BRL, Grand Island, NY, USA) containing 5% newborn calf serum (NCS, GIBCO BRL) with red phenol supplemented by benzylpenicillin. Cultures were maintained in a humidified atmosphere with 5% CO_2_ at 37 °C. All cell-based assays were performed using cells in the exponential growth phase.

#### 3.3.2. Cell Proliferation Assays

Assays were performed by seeding 7500 cells/well in 96-well tissue culture plates at a volume of 100 μL of RPMI-1640 medium supplemented with 5% NCS per well. Cells were allowed to grow for 24 h in the culture medium prior to exposure to the compounds. Cisplatin was used as positive control. Additionally, 1% of vehicle (DMSO/EtOH, 1:1) was added to the control cells. Antiproliferative activity (IC_50_) was determined after 24 h by crystal violet staining [71]. Cell counts were performed by measuring absorbance at 590 nm in an ELISA plate reader (ChroMate, Palm, FL, USA).

#### 3.3.3. Determination of Cytotoxicity

Cytotoxic activity was determined by means of LDH Cytotoxicity Assay Kit (Cyto Tox 96^®^ Promega, Madison, WI, USA) following the manufacturer’s instructions. LDH oxidizes lactate to pyruvate, which then reacts with the INT to produce formazan. The increased amount of formazan generated in the culture supernatant directly correlates to the increase in the number of lysed cells. Formazan dye is water-soluble and can be detected with a spectrophotometer at 500 nm [51].

#### 3.3.4. CFSE-Labeling Assay

Heparinized blood samples were obtained from healthy human volunteers. PBMCs were isolated using standard Hypaque (Sigma-Aldrich, St. Louis, MO, USA) density gradient centrifugation. The PBMCs were then washed twice with RPMI 1640 (GIBCO BRL) medium containing 10% NCS, penicillin (100 U/mL), and streptomycin (100 μg/mL). The lymphocyte population was further enriched (ELP) by eliminating adherent cells (cells were incubated at 37 °C, 5% CO_2_ for 1 h, and non-adherent cells were harvested). ELPs were re-suspended in RPMI-1640 medium at a concentration of 1 × 10^6^ cells/mL. CFSE (Sigma-Aldrich) was added to the cell suspension at a final concentration of 2 mM and then incubated for 15 min at room temperature in the dark. Labeling was completed by adding the same volume of NCS during 5 min at room temperature to quench the free CFSE. Labeled cells were washed five times with sterile PBS containing 10% NCS, counted, and then re-suspended in RPMI-1640 medium at 1 × 10^6^ cells/mL [72]. Unstimulated, PHA-stimulated, or treated, cells were plated at 2 × 10^5^ cells/well in 96-well, flat-bottomed cell culture plates, and five replicate samples for each treated amount were prepared. Cells were incubated in a 5% CO_2_ incubator at 37 °C for 72 h. Cultured cells were harvested, washed twice with PBS, fixed with 1% formaldehyde, then analyzed by flow cytometry, acquiring a minimum of 20,000 events from each sample. Data analysis was performed using FACSDiva software (4.0, Piscataway, NJ, USA).

#### 3.3.5. Detection of Active Caspase-3

HeLa and CaSki cells were seeded at 10^5^ cells/mL in 60-mm tissue culture plates and allowed to grow for 24 h in culture medium before treatment with their respective IC_50_. Cells were harvested with versene solution. For active caspase-3 immunodetection, they were fixed and permeabilized in 50% methanol in PBS, washed in PBS, incubated with primary anti-body anti-active-caspase-3 (ABCAM, Cambridge, MA, USA), and diluted in PBS (1:500) for 18 h. Next, the cells were washed and incubated with the secondary goat anti-rabbit antibody with FITC diluted to 1:200 in PBS for 2 h. The samples were analyzed by flow cytometry, acquiring a minimum of 10,000 events from each sample. Data analysis was performed using FACSDiva 4.0 software.

#### 3.3.6. Statistical Analysis

Means and standard deviations (SD) were calculated using Excel (Microsoft Office, Version 2010, Redmond, WA, USA). Statistical analysis of differences was carried out by ANOVA using SPSS 10.0 for Windows. A *p*-value < 0.05 (Student’s *t*-test) was considered significant.

## 4. Conclusions

Two new steroidal sapogenins were synthesized, with the incorporation of the functional group oxime to measure their antiproliferative activity, in the search for compounds with an increased broad biological activity profile. The assays showed that the steroidal oximes **4** and **5** generate interesting results in the antiproliferative activity in vitro compared to the activity observed with diosgenin (**1**). The antiproliferative activity of **4** was 2.3–2.8 times that of **1** due to the oxime group present in the spiroketalic side chain of **4**; the oxime groups present on the steroidal A/B rings of **5** decreased the antiproliferative activity 1.2–1.7 times, suggesting that the oxime group improves the activity of spirostans if it is present in the side chain. A relevant aspect of compounds **4** and **5** is that they produce low-cytotoxicity effects in two cellular types: cancer cells and normal cells (lymphocytes), since apoptosis, more than necrosis, was the pathway for cancer cell elimination with evidenced apoptotic morphology and an increased presence of active caspase-3. The fact that compounds **4** and **5** did not affect proliferation of normal lymphocytes confers a certain advantage to these compounds. It has been shown that the double bond between positions C4 and C5 on the steroidal ring A, confers a negative cytotoxic effect on cancer cells for 3-hydroxyimino-substituted compounds [35]. This suggests that antiproliferative activity may also be mediated, at least to some extent, by a different behavior between steroidal oximes **4** and **5**, related to the conformational difference on the rings A/B (a double bond shift) and it is possible that compound **4** may be recognized more easily by the active site in the cell by binding to protein- associated signaling molecules [73]. From this information our future work will focus on the search for new routes to synthesize steroidal derivatives with oxime moieties on a larger scale and on the search for molecular mechanisms involved in antiproliferative activity. The information obtained may be useful for the design of novel chemotherapeutic drugs.

## Figures and Tables

**Table 1:** IC_50_ values of compounds **1**, **4**, and **5** on cervicouterine cancer cell lines HeLa and CaSki.

Cell line	IC_50_ μg/ML (μM)		
	Diosgenin (1)	Oxime (4)	Dioxime (5)
HeLa	15 (36.17)	5.6 (10.9)	19 (41.61)
CaSki	13 (31.35)	5.4 (10.51)	22 (48.18)

## Supplementary Materials

Supplementary materials can be accessed at: http://www.mdpi.com/1420-3049/21/11/1533/s1, Figure S1–Figure S22.
