# Supplementary material for: Antiproliferative, Cytotoxic, and Apoptotic Activity of Steroidal Oximes in Cervicouterine Cell Lines
Source: Molecules. 2016 Nov 14;21(11):1533. doi: 10.3390/molecules21111533 (PMC6273349; doi:10.3390/molecules21111533)
Supplement: Supplementary file 1 [file molecules-21-01533-s001.pdf]

Supplementary Materials: Antiproliferative, Cytotoxic, and Apoptotic Activity of Steroidal Oximes in Cervicouterine Cell Lines

Luis Sánchez-Sánchez, María Guadalupe Hernández-Linares, María L. Escobar, Hugo López-Muñoz, Edgar Zenteno, María A. Fernández-Herrera, Gabriel Guerrero-Luna, Alan Carrasco-Carballo and Jesús Sandoval-Ramírez

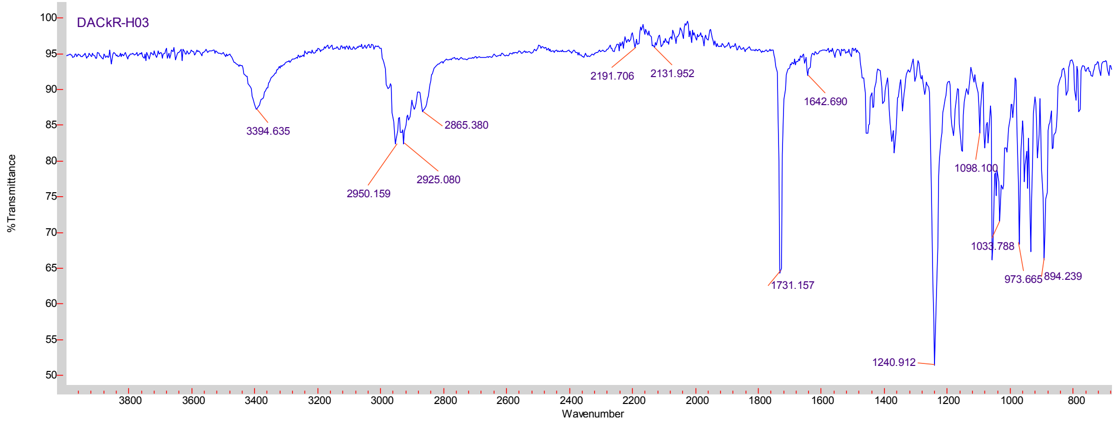

Figure S1. IR spectrum of the oxime of (22S,23*R*)-acetyldiosgenin acetate (**4**).

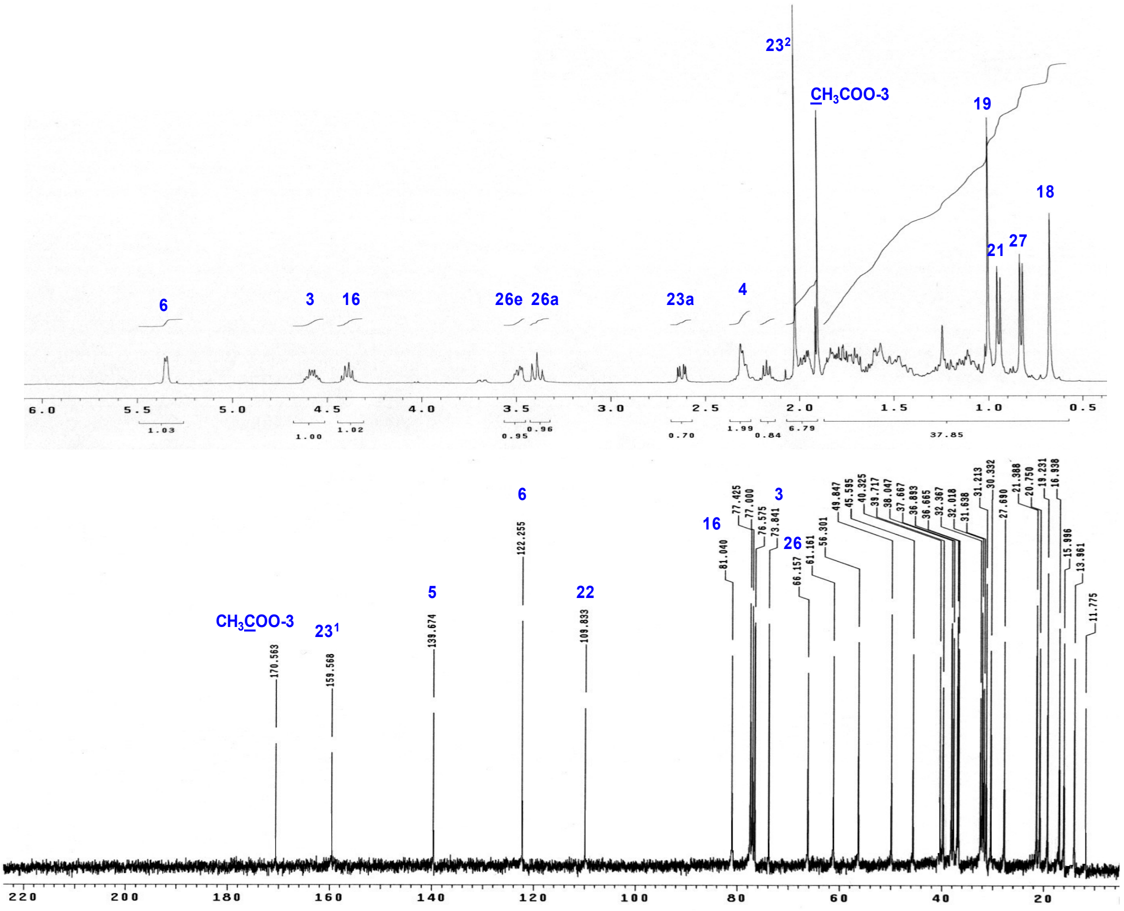

Figure S2. ^{1}H-NMR and ^{13}C-NMR spectra of the oxime of (22S,23*R*)-acetyldiosgenin acetate (**4**).

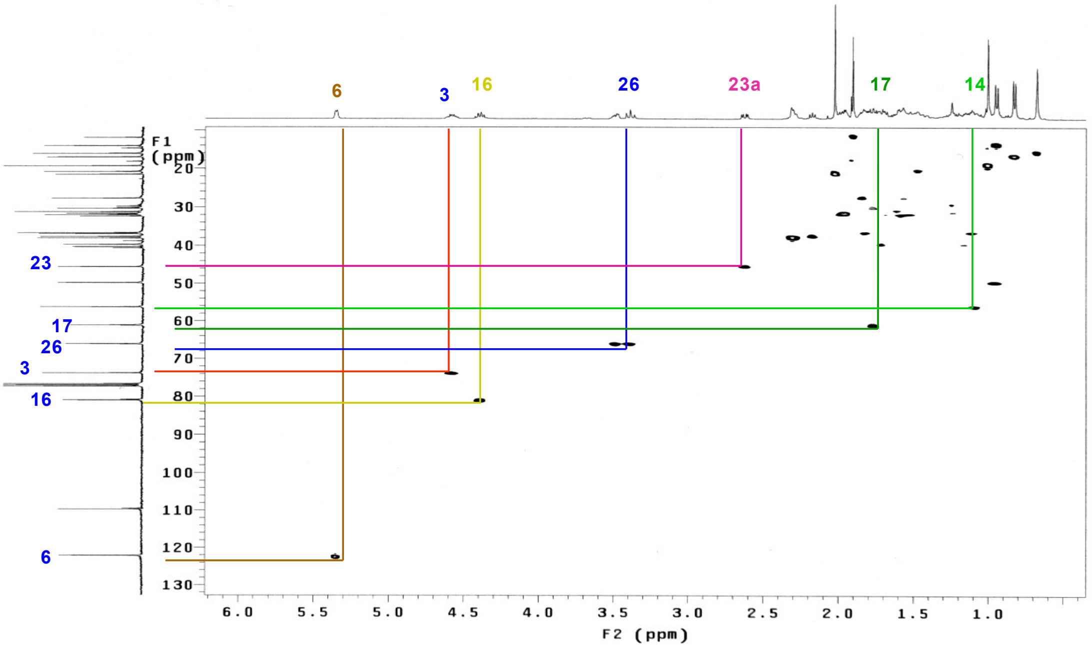

Figure S3. HSQC experiment of the oxime of (22S,23*R*)-acetyldiosgenin acetate (**4**).

[Elemental Composition] Page: 1
Data : Dr-Cuevas-Gabriel021 Date : 28-Jun-2016 18:36
Sample: 2556 MG54D JeolSX102A
Note : Operadores Carmen Garcia javier Perez
Inlet : Direct Ion Mode : EI+
RT : 1.13 min Scan#: (7,10)
Elements : C 40/0, H 69/0, O 7/3, N 2/1
Mass Tolerance : 1000ppm, 3mmu if m/z < 3, 5mmu if m/z > 5
Unsaturation (U.S.) : -0.5 - 15.0

Observed m/z	Int%
513.3445	10.6

Estimated m/z	Error[ppm]	U.S.	C	H	O	N
513.3454	-1.7	9.0	31	47	5	1

Figure S4. Data from the high resolution mass spectrum of the oxime **4**.

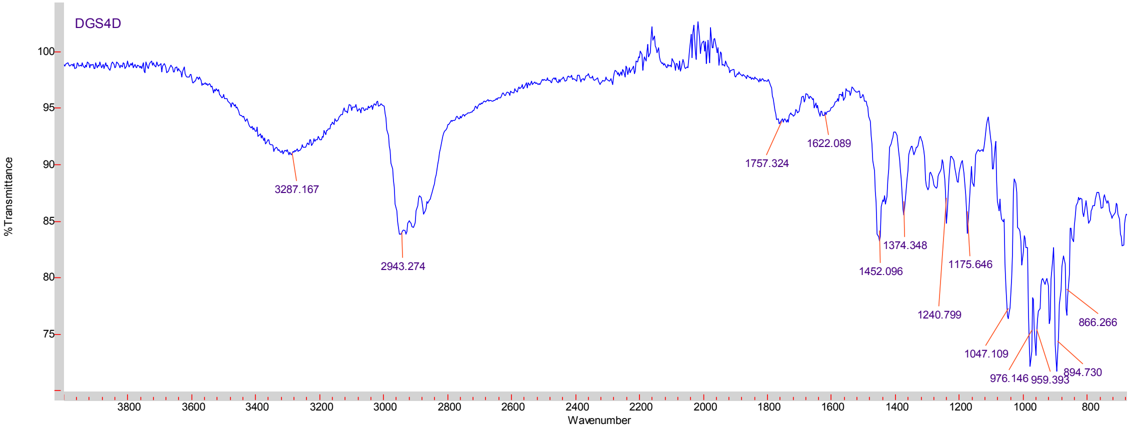

Figure S5. IR spectrum of the dioxime of (22*S*,25*R*)-spirost-4-en-3,6-dione (**5**).

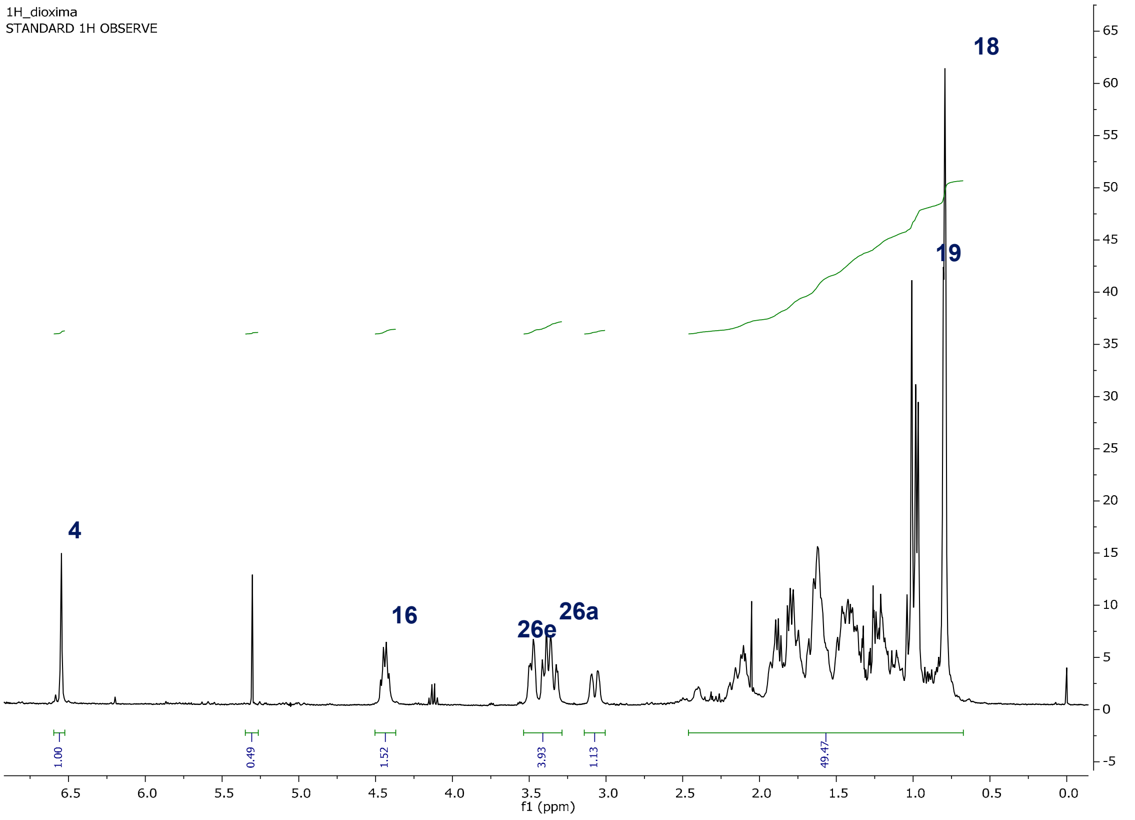

Figure S6. ^{1}H-NMR spectrum of the dioxime of (22*S*,25*R*)-spirost-4-en-3,6-dione (**5**).

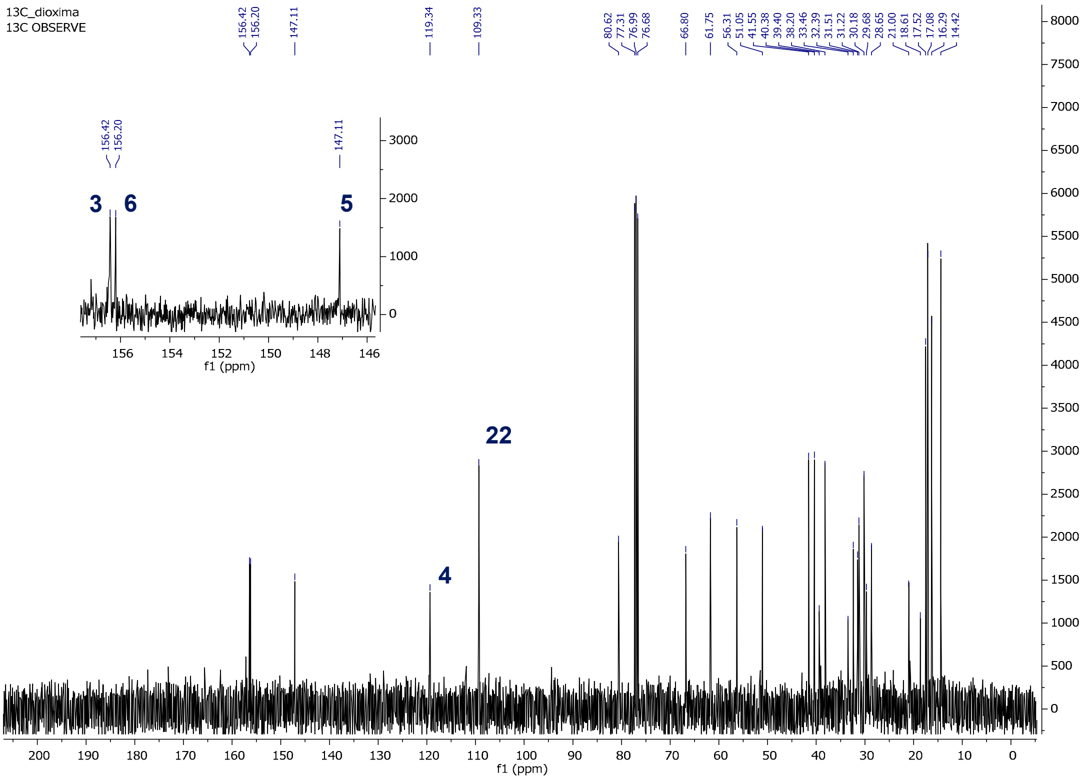

Figure S7. ^{13}C-NMR spectrum of the dioxime of (22*S*,25*R*)-spirost-4-en-3,6-dione (**5**).

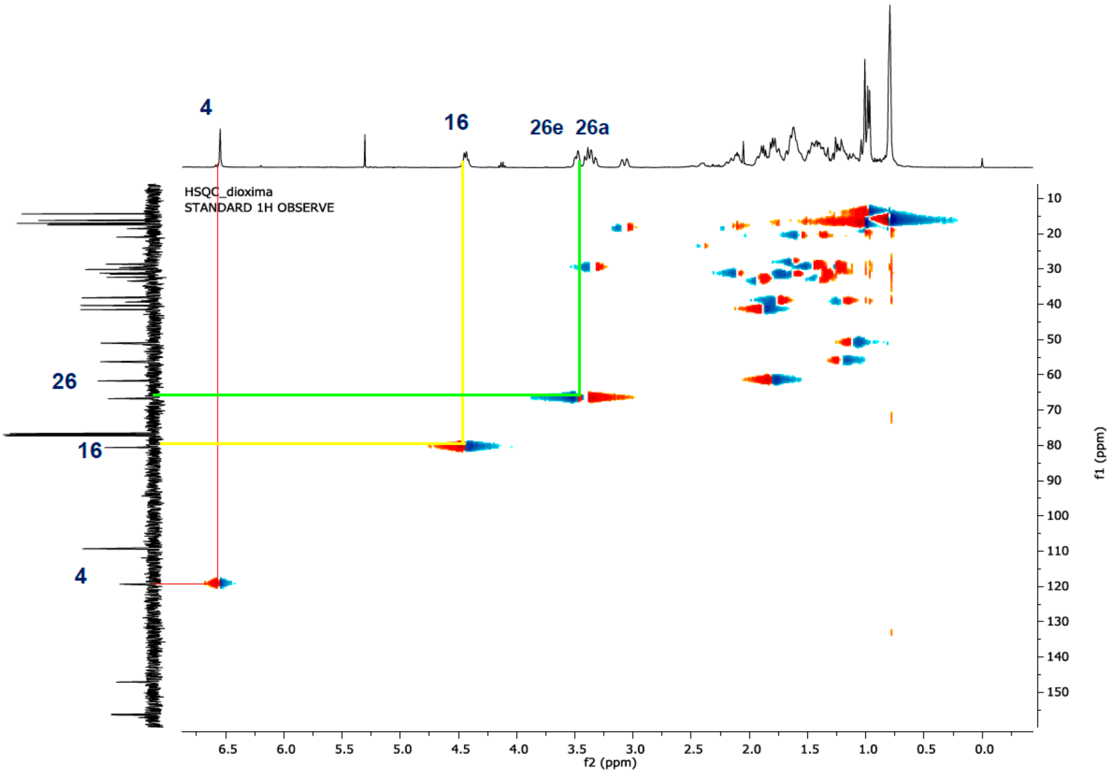

Figure S8. HSQC experiment of the dioxime of (22*S*,25*R*)-spirost-4-en-3,6-dione (**5**).

[Elemental Composition] Page: 1
Data : Dr-Cuevas-Gabriel020 Date : 28-Jun-2016 18:30
Sample: 2555 DG54D JeolSX102A
Note : Operadores Carmen Garcia javier Perez
Inlet : Direct Ion Mode : EI+
RT : 0.98 min Scan#: (6,9)
Elements : C 40/0, H 69/0, O 5/2, N 3/1
Mass Tolerance : 1000ppm, 3mmu if m/z < 3, 5mmu if m/z > 5
Unsaturation (U.S.) : -0.5 - 15.0

Observed m/z	Int%
456.2982	75.0

Estimated m/z	Error [ppm]	U.S.	C	H	O	N
456.2988	-1.2	9.0	27	40	4	2

Figure S9. Data from the high resolution mass spectrum of the dioxime **5**.

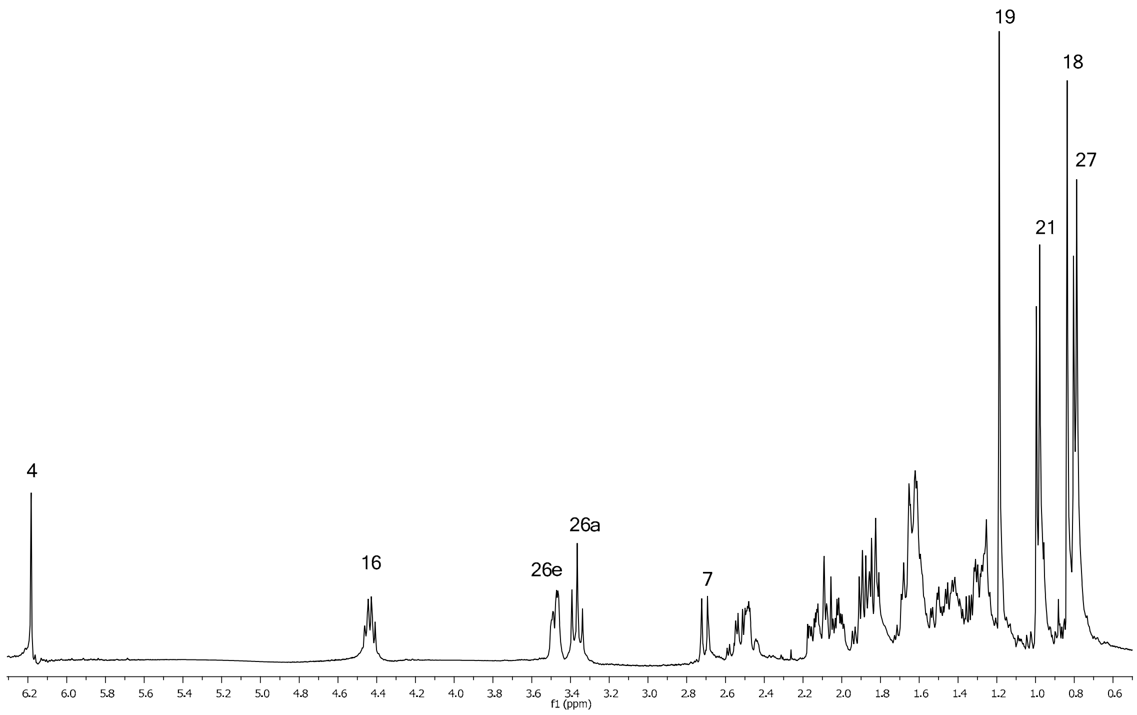

Figure S10. ^{1}H-NMR spectrum (500 MHz) of (22*S*,25*R*)-spirost-4-en-3,6-dione **9**.

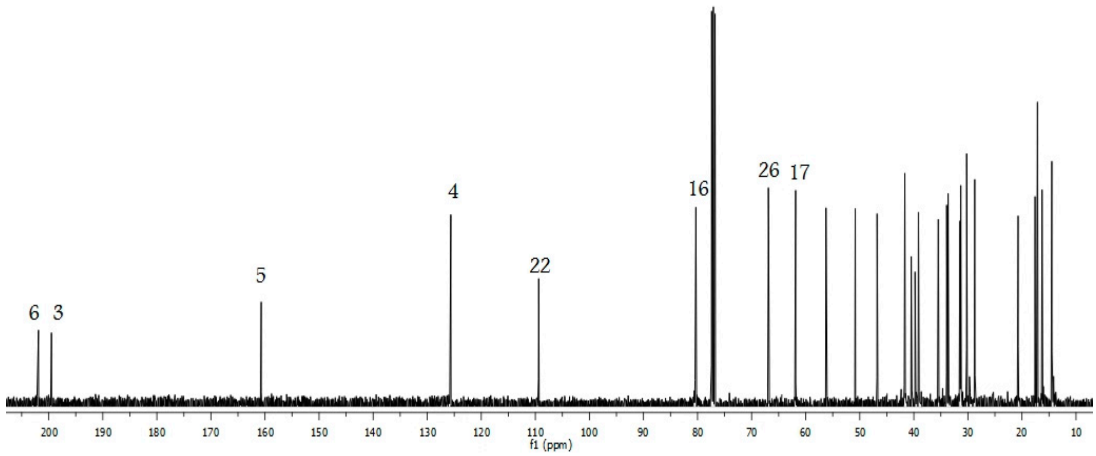

Figure S11. ^{13}C-NMR spectrum (125 MHz) of (22*S*,25*R*)-spirost-4-en-3,6-dione **9**.

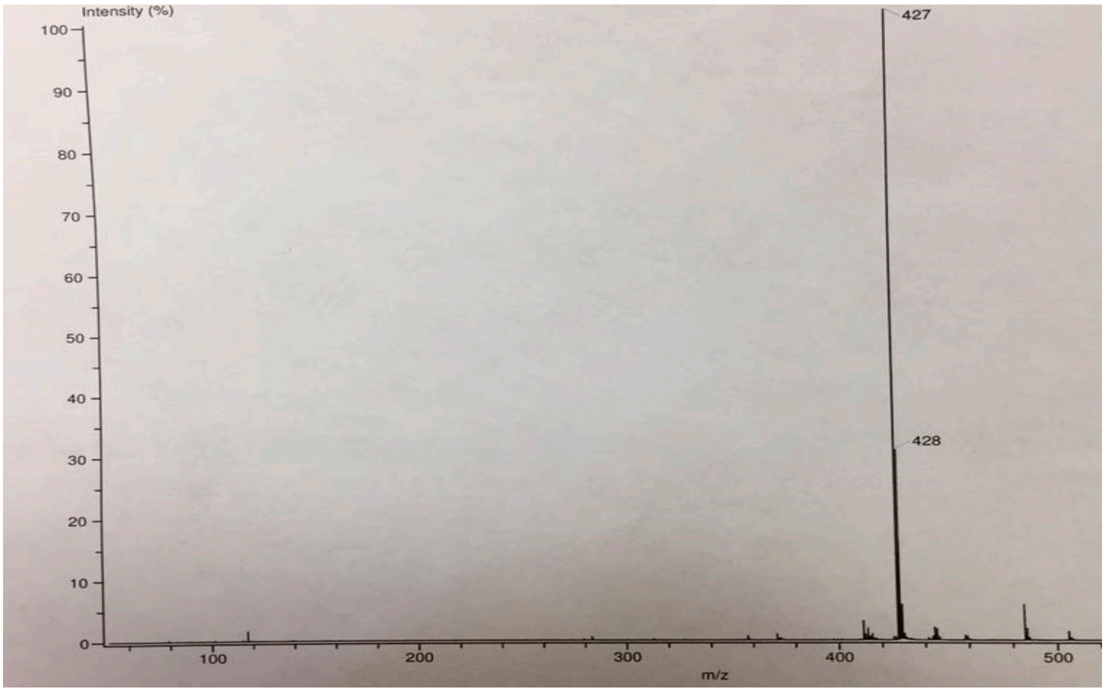

Figure S12. MS of (22*S*,25*R*)-spirost-4-en-3,6-dione **9**.

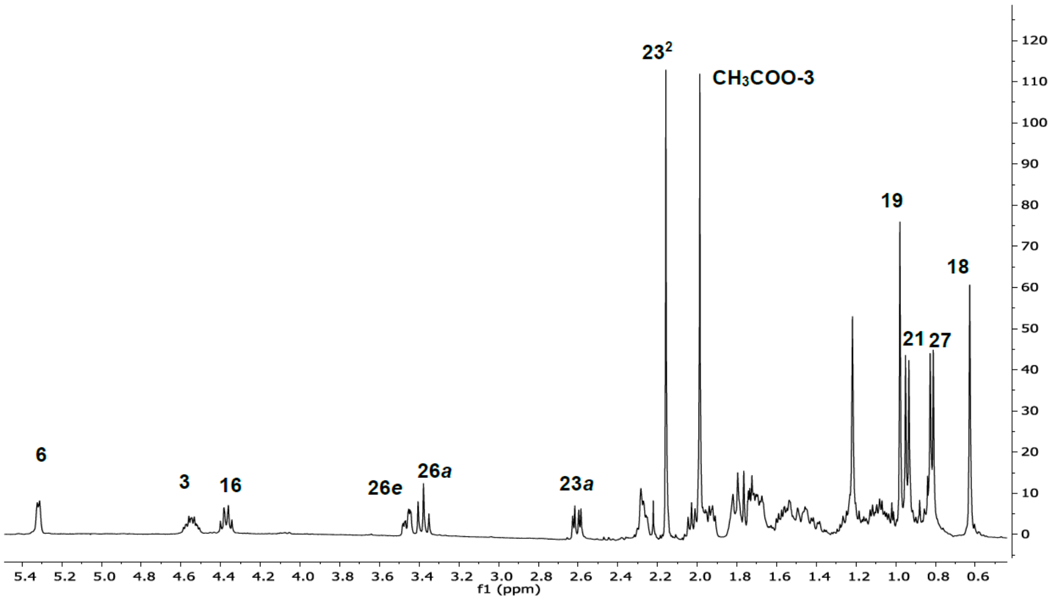

Figure S13. ^{1}H-NMR spectrum (500 MHz) of (23*R*)-acetyldiosgenin acetate **8**.

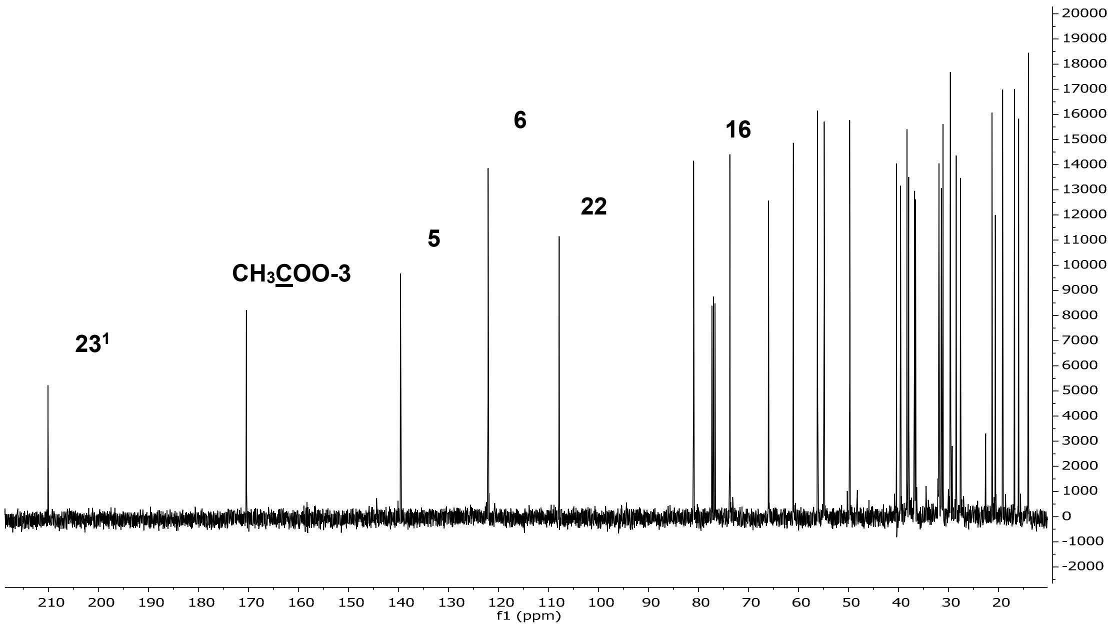

Figure S14. ^{13}C-NMR spectrum (125 MHz) of (23*R*)-acetyldiosgenin acetate **8**.

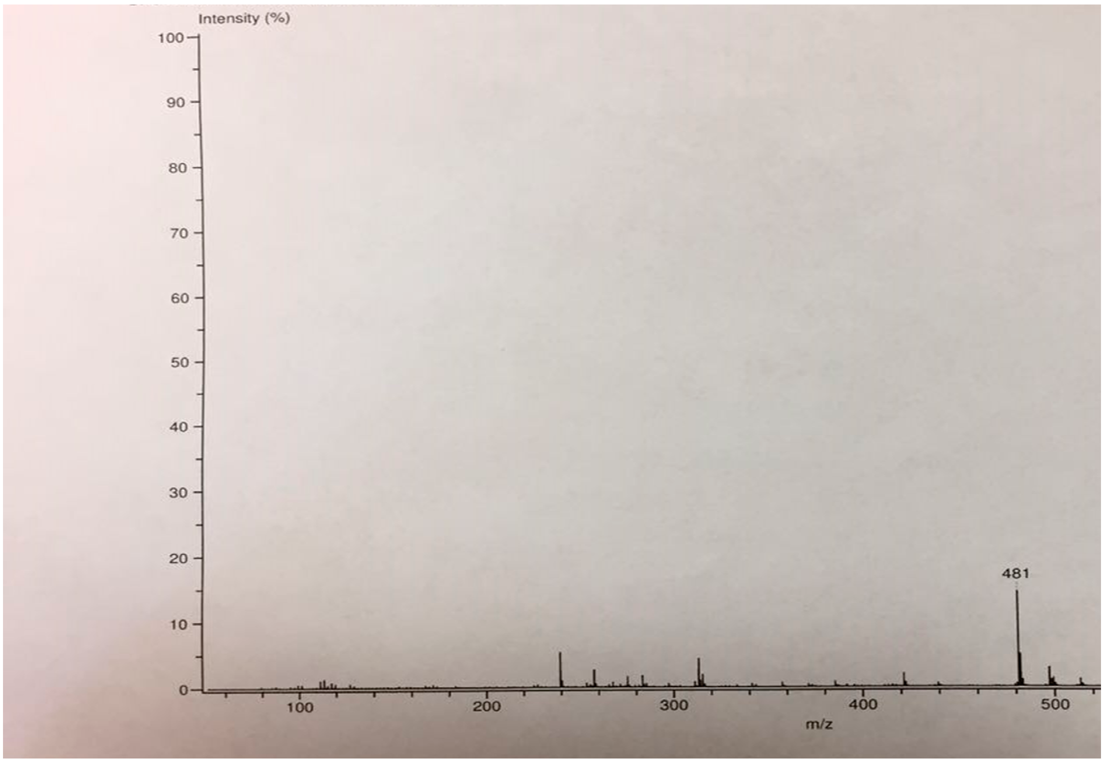

Figure S15. MS of (23*R*)-acetyldiosgenin acetate **8**.

Minimum energy structures of the *anti* isomer (Figure S16), and *syn* isomer (Figure S17). Figure S17 shows the *syn* isomer, where CH_3-23^2 has a distance of 4.22 Å on average, with respect to CH_3-21, by free rotation; so we would expect to observe a NOE-effect when irradiated CH_3-23^2 expecting to affect the CH_3-21, which is not observed (see Figure S18). However, when the case of the *anti* isomer is analyzed, the distance from CH_3-21 to CH_3-23^2 has a distance of 6.45 Å, which would not allow a direct NOE-effect and not would be observed when irradiated with CH_3-23^2, as it was established in this experiment.

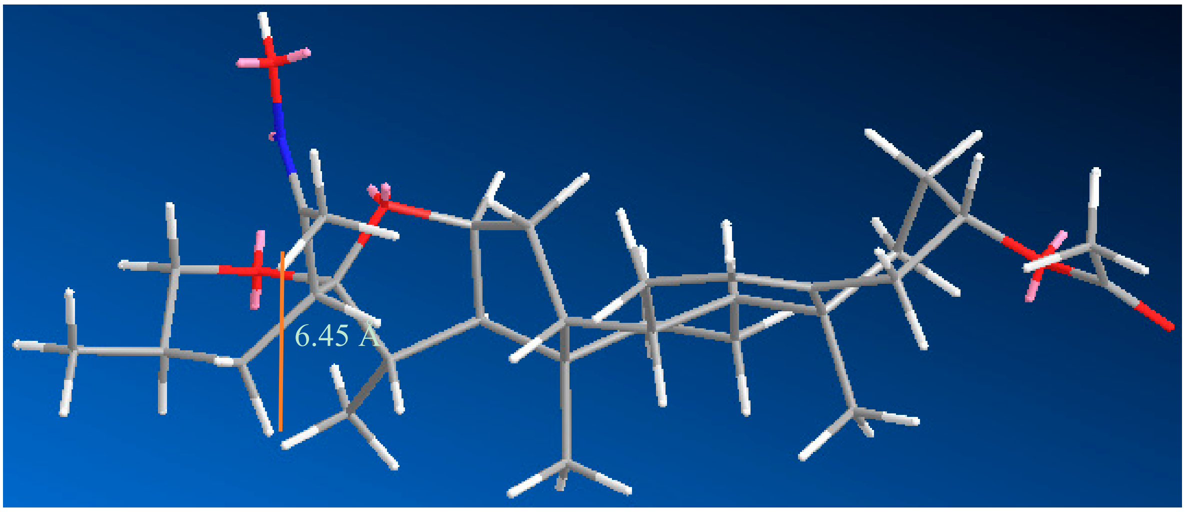

Figure S16. Structure optimized (MM2) of *anti* isomer of oxime **4**, and distance through space between CH_3-21 and CH_3-23^2.

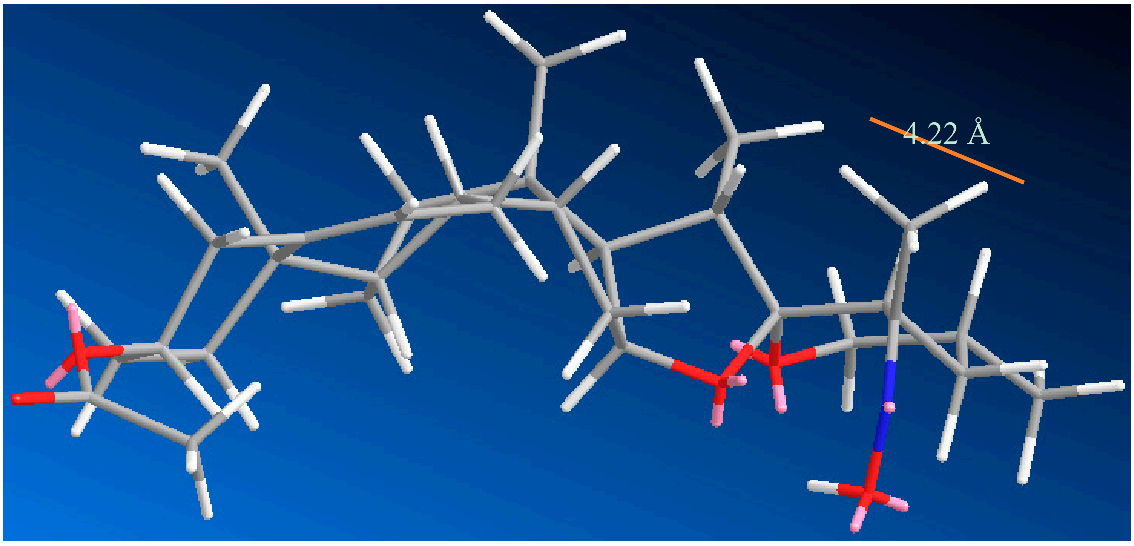

Figure S17. Structure optimized (MM2) of hypothetic *syn* isomer of oxime **4**, and distance through space between CH_3-21 and CH_3-23^2.

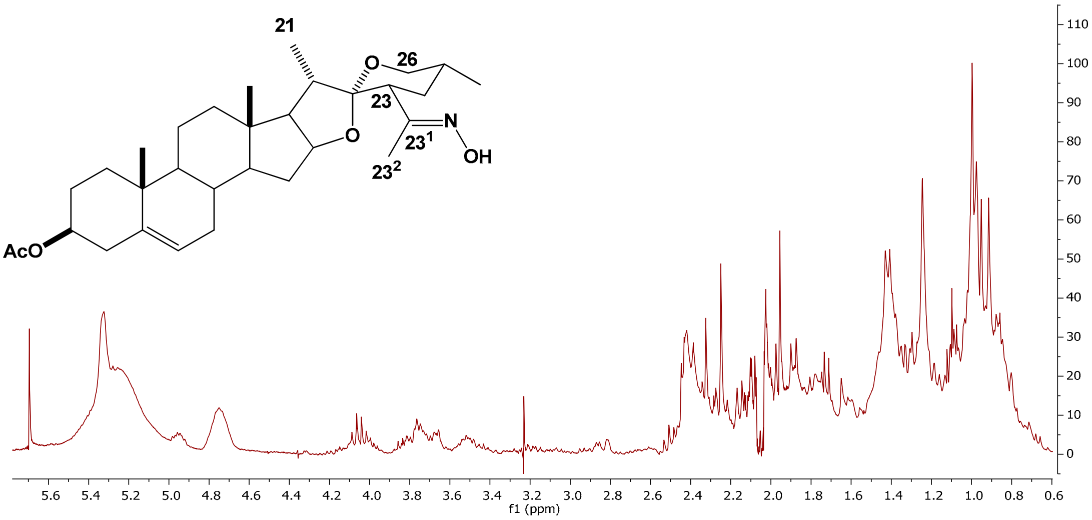

Figure S18. NMR-NOE experiment (300 MHz) of the protons CH_3-23^2 that show only affects H-26*eq*.

A NOE-effect was observed around 3.3 ppm when CH_3-23^2 is irradiated, due to the distance at the H-26*eq* (4.5 Å, see Figure S19); however, in the geometric isomer *syn* (Figure S20), the distance to this proton from CH_3-23^2 corresponding to 5.77 Å, so one should not observe this phenomenon, thus demonstrating that the geometry of the oxime **4** is *anti*.

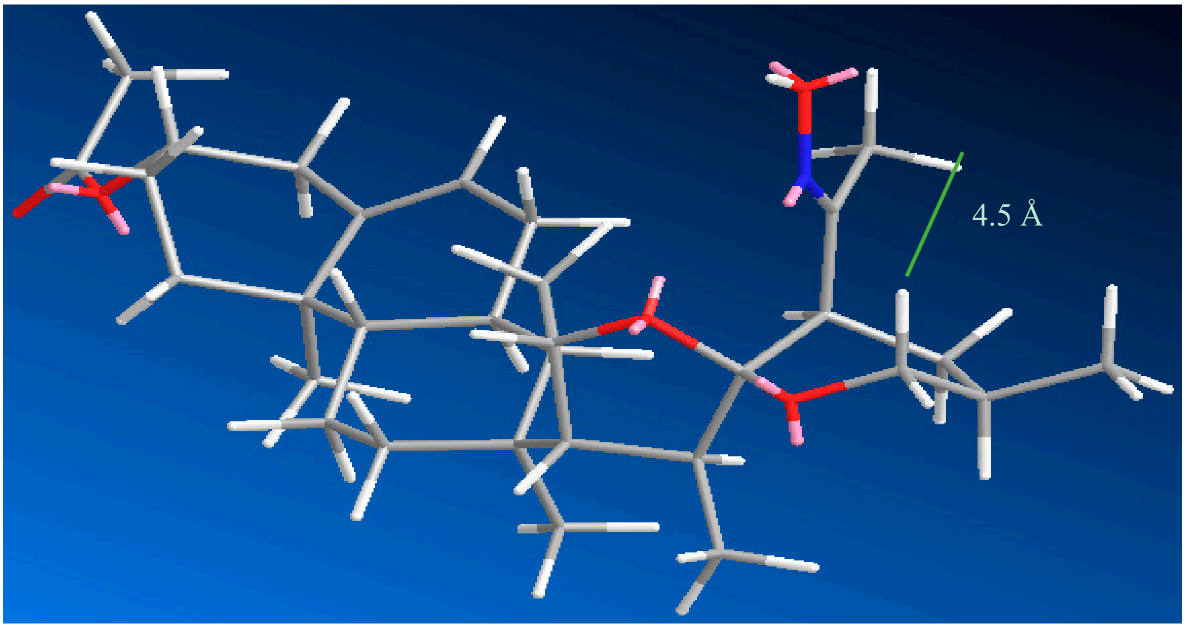

Figure S19. Structure optimized (MM2) of *anti* isomer of oxime **4**, and distance through space between CH_3-21 and H-26*eq*.

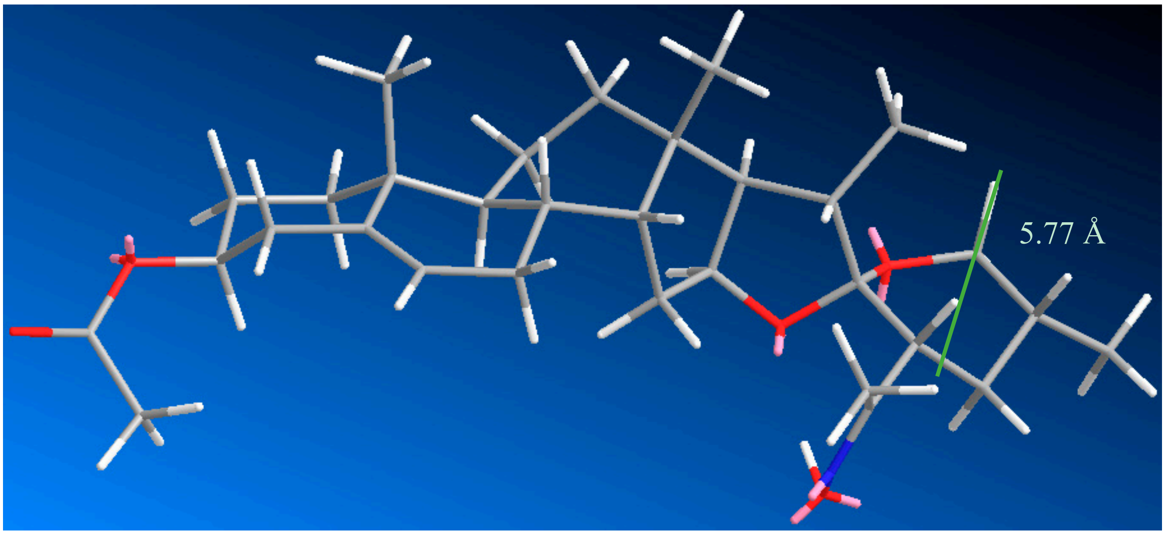

Figure S20. Structure optimized (MM2) of hypothetic *syn* isomer of oxime **4**, and distance through space between CH_3-23^2 and H-26*eq*.

The NOESY experiment was performed (500 MHz), in Figure S21 shows that there is no correlation between CH_3-23^2 and CH_3-21, as previously described above. Additionally, in Figure S22, the correlation is confirmed through space between CH_3-23^2 and H-26*eq*.

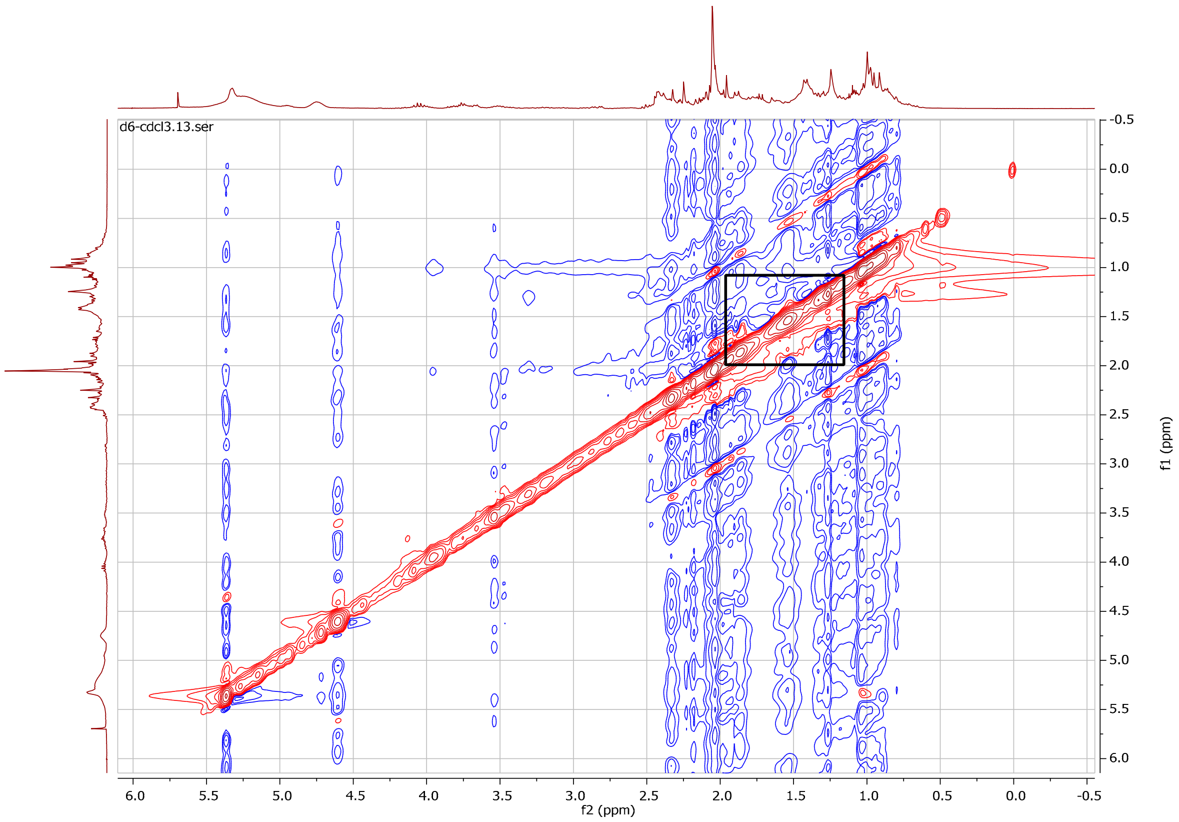

Figure S21. NMR-NOESY spectrum (500 MHz) of oxime **4**, which shows that there is no correlation through space between CH_3-23[2] to CH_3-21.

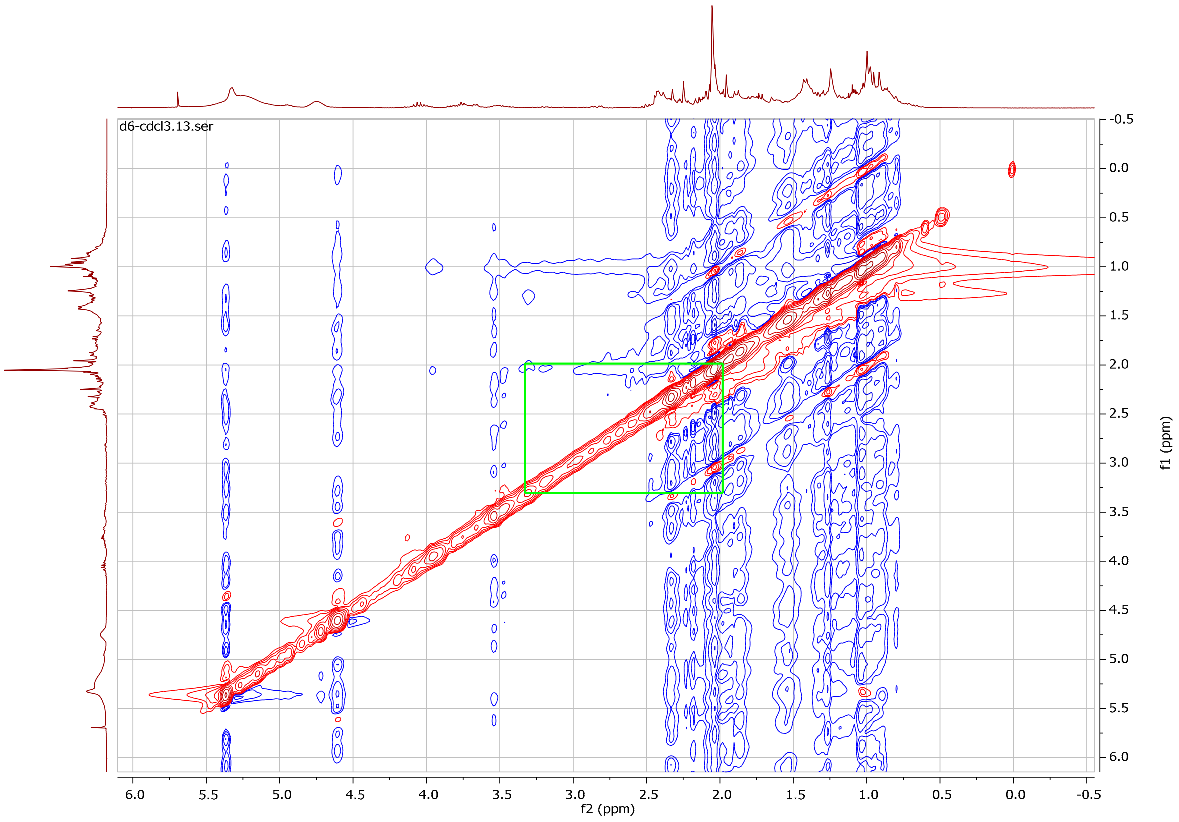

Figure S22. NMR-NOESY spectrum (500 MHz) of oxime **4**, which shows that there is no correlation through space between CH_3-23[2] to H-26*eq*.
